# Supplementary material for: Ocean warming drives rapid dynamic activation of marine-terminating glacier on the west Antarctic Peninsula
Source: Nat Commun. 2023 Nov 28;14:7535. doi: 10.1038/s41467-023-42970-4 (PMC10684579; doi:10.1038/s41467-023-42970-4)
Supplement: Supplementary file 1 — Supplementary Information [file 41467_2023_42970_MOESM1_ESM.pdf]

Supplementary online material for: Ocean warming drives rapid dynamic activation of marine-terminating glacier on the west Antarctic Peninsula

Benjamin J. Wallis[1], Anna E. Hogg[1], Michael P. Meredith[2], Romilly Close[3], Dominic Hardy[3], Malcolm McMillan[3], Jan Wuite[4], Thomas Nagler[4], Carlos Moffat[5]

[1]Institute for Climate and Atmospheric Science, University of Leeds, Leeds, United Kingdom

[2]British Antarctic Survey, Cambridge, United Kingdom

[3]UK Centre for Polar Observation & Modelling, Centre of Excellence in Environmental Data Science, Lancaster Environment Centre, Lancaster University, Lancaster, United Kingdom

[4]ENVEO IT GmbH, Innsbruck, Austria

[5]School of Marine Science and Policy, University of Delaware, Newark, Delaware, United States

Correspondence to: Benjamin J. Wallis (eebjwa@leeds.ac.uk)

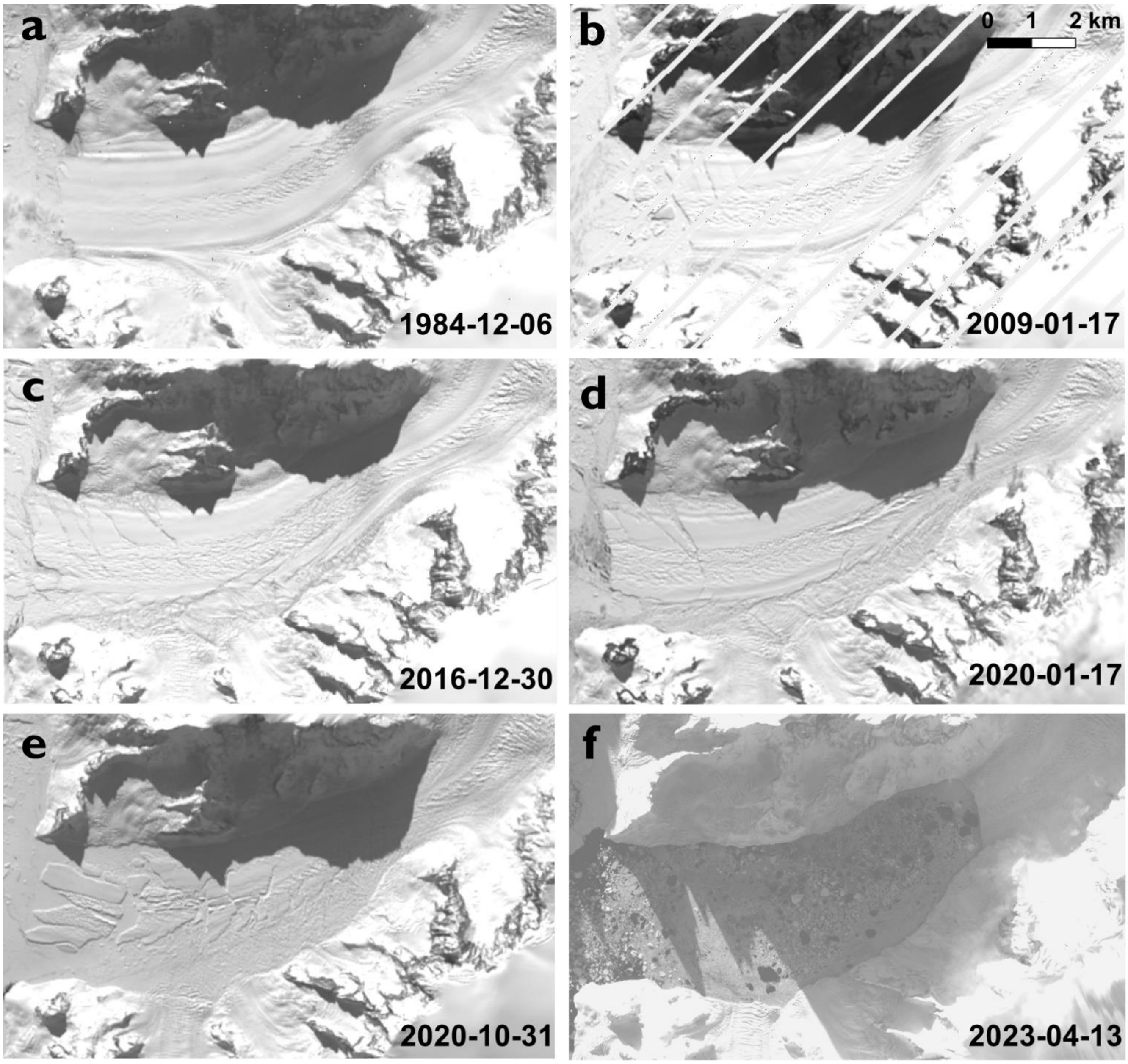

Figure S1 – Damage on Cadman Glacier. Change in damage on the surface of Cadman Glacier Ice Shelf from 1984 to 2023. Panels a-f show greyscale Landsat-5, -7, -8, -8 -8 and Copernicus Sentinel-2 images of Cadman Glacier Ice Shelf, showing the pattern of increasing damage over the study period and the collapse of the ice shelf.

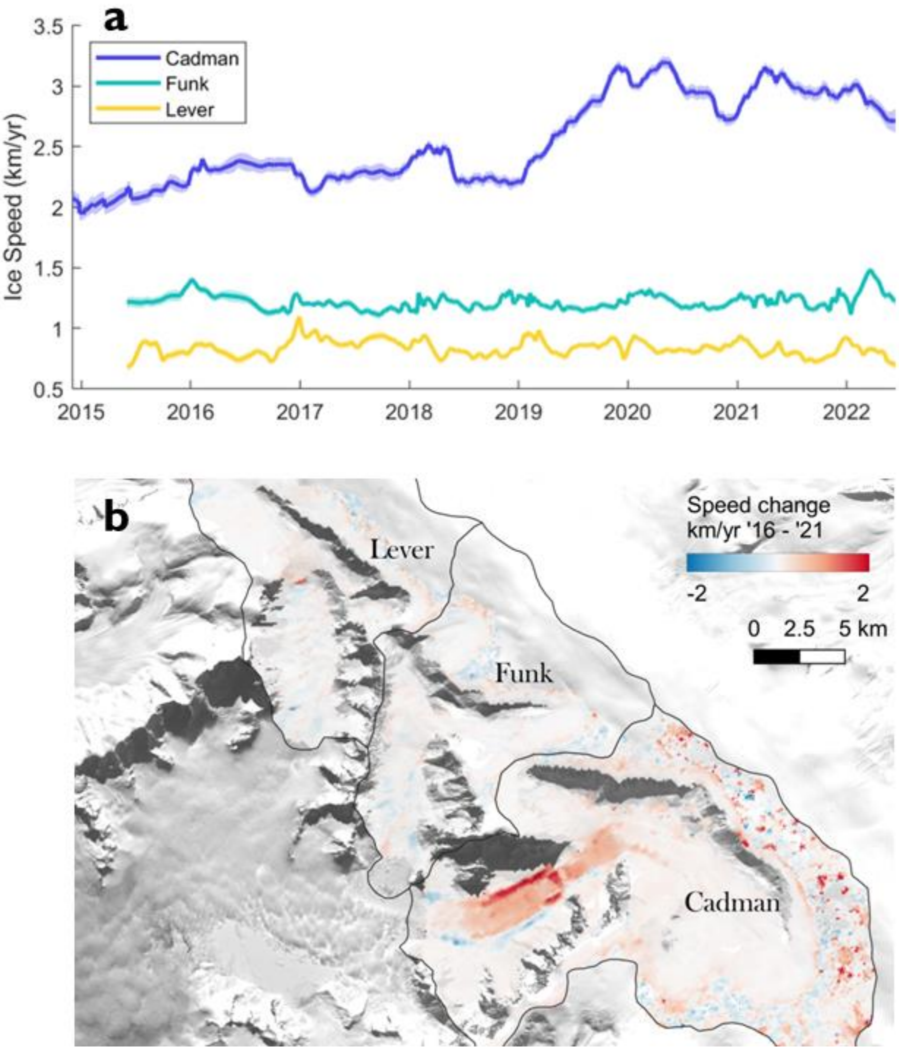

Figure S2 - Speed time-series and speed change map for Cadman, Funk and Lever glaciers. (a) Ice speed time-series: Ice speed is extracted over the lower glacier grounded ice sample area on Cadman Glacier defined in Fig. 1b and Fig. 3b. On Funk and Lever Glaciers ice speed is extracted at a point 1 km inland of the calving front location, which has remained stable throughout the study period. Shading on all time-series shows an uncertainty of one standard deviation. (b) Speed change map calculated between annual means for 2016 and 2021. Glacier drainage basins are labelled in black[1]. Base image is Landsat-5 6th December 1984.

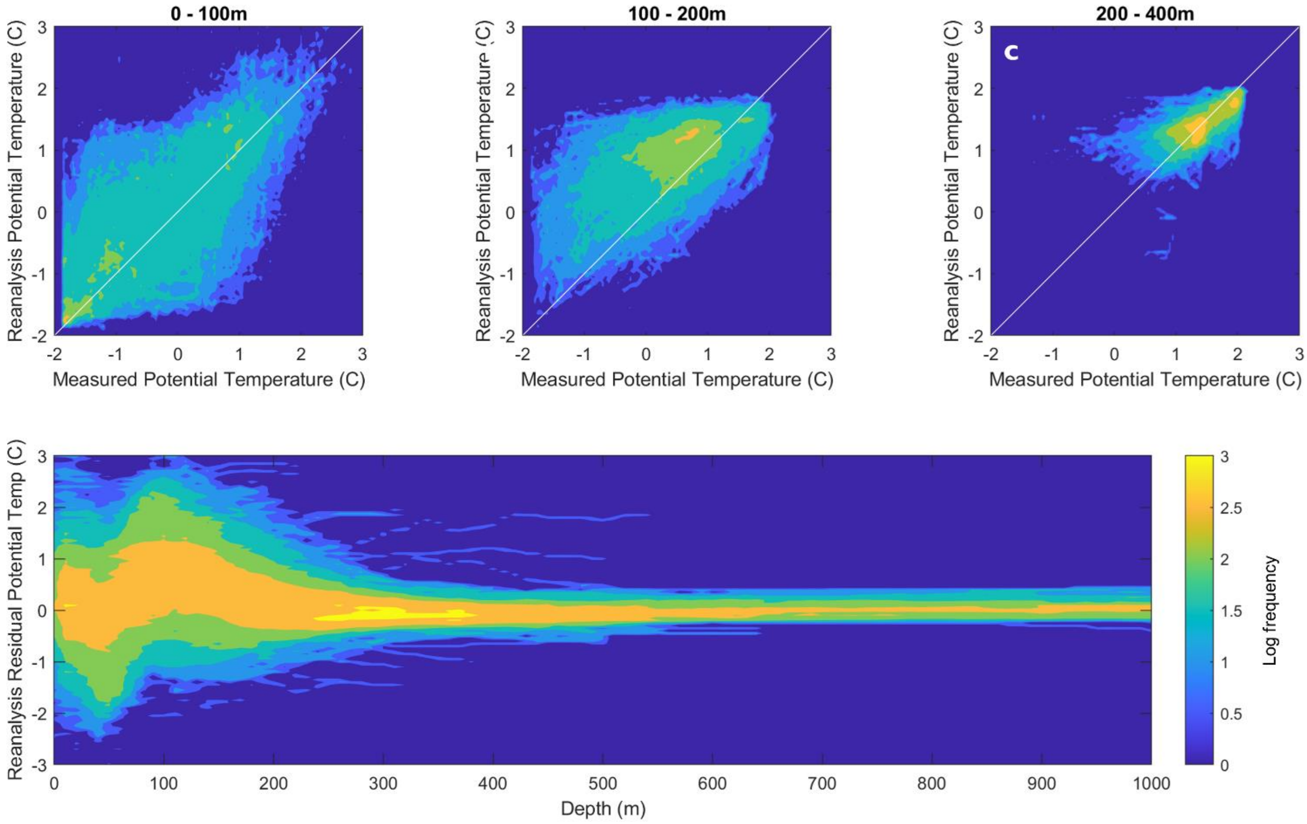

Figure S3 – Ocean temperature data intercomparison. Comparison of GLORYS global ocean physical reanalysis model[2] and Palmer Long-Term Ecological Research programme oceanographic station in-situ measurements at 0 to -100 m, -100 to -200 m, -200 to -400 m depth intervals (a-c respectively) vertically through the water column, with the residuals shown from 0 to -1000 m throughout the water column (d).

Supplementary References

1. Cook, A. J., Vaughan, D. G., Luckman, A. J. & Murray, T. A new Antarctic Peninsula glacier basin inventory and observed area changes since the 1940s. *Antarctic Science* **26**, 614–624 (2014).
2. Jean-Michel, L. *et al.* The Copernicus Global 1/12° Oceanic and Sea Ice GLORYS12 Reanalysis. *Frontiers in Earth Science* **9**, 585 (2021).
